# Prostaglandin F2α agonists induced enhancement in collagen1 expression is involved in the pathogenesis of the deepening of upper eyelid sulcus

**DOI:** 10.1038/s41598-021-88562-4

**Published:** 2021-04-26

**Authors:** Kaku Itoh, Yosuke Ida, Hiroshi Ohguro, Fumihito Hikage

**Affiliations:** Departments of Ophthalmology, Sapporo Medical University School of Medicine, Sapporo, Japan

**Keywords:** Eyelid diseases, Ocular hypertension, Optic nerve diseases

## Abstract

Previous our study reported that three-dimension (3D) cultures of human orbital fibroblasts (HOFs) replicated the etiology of deepening of the upper eyelid sulcus (DUES) caused by prostaglandin F2α analogues (PGF2α-ags). To examine this further, the effects of PGF2α-ags on HOFs were characterized by (1) lipid staining (2D; two-dimension, 3D), (2) comparison of the 3D organoid sizes of preadipocytes (DIF−) or adipocytes (DIF+) that had been treated with various concentrations of several PGF2α-ags, (3) physical stiffness (3D), and (4) the mRNA expression of adipogenic related genes, extracellular matrix (ECM), tissue inhibitors of metalloproteinases (TIMPs) and matrix metalloproteinases (MMPs) (3D). PGF2α-ags caused a dramatic down-sizing of the 3D DIF+ organoids and this reduction was concentration dependent. The effects caused by PGF2α-ags were also observed in 3D preadipocytes. Micro-squeezer analysis clearly indicated that PGF2α-ags induced an increase in their physical solidity. The size of each organoid under several conditions was inversely correlated with the mRNA expression profile of *collagen1 (COL1)*, *TIMP2, and MMP2* and *9*. These findings indicate that PGF2α-ags affect the expression of *COL1*, *TIMP2*, and *MMP2* and *9* which, in turn, modulate the 3D ECM network within the organoids, thus resulting in their downsizing.

## Introduction

Prostaglandins (PGs) exert their effects via paracrine and autocrine on target cells by coupling to their specific G-protein coupled receptors and activating intracellular signaling^1,2^. Among the PGs, PGF2α, PGE2 and PGI2 are the most abundantly biosynthesized and are the major metabolites of cyclooxygenase (COX) enzymes^1,2^. PGF2α analogues (PGF2α-ags) are typically used as first-line drugs because of their efficiency in lowering elevated intraocular pressure (IOP) through their FP receptors as well as not causing serious systemic side effects^3–5^. It should be noted in this respect that, among long-term users of PGF2α-ags, local side effects that are referred to as “prostaglandin-associated periorbitopathy (PAP)” including deepening of the upper eyelid sulcus (DUES), conjunctival injection, hyperpigmentation of the iris and skin and elongation of the eyelashes have recently been reported^6–8^. Among these, DUES is a non-negligible cosmetic side effect, and has been reported for most PGF2α-ags including bimatoprost (BIM), travoprost, tafluprost, and latanoprost (LAT)^9–12^. As possible mechanisms responsible for causing DUES, orbital fat atrophy, as observed by MRI and histological analysis appears to be primarily involved^13^. However, the precise molecular etiology remains to be elucidated.

In terms of the molecular etiology of DUES, several previous reports using two-dimension (2D) cell cultures have revealed that PGF2α-ags suppress adipogenesis through the activation of the FP receptor^14^. Thus, such PGF2α-ags induced suppression of the adipogenic differentiation of adipocytes may primarily be involved in the pathogenesis causing DUES. Furthermore, in our previous study, to establish a suitable model for mimicking the etiology of DUES by PGF2α-ags, we used tissue cultures of 3T3-L1 cells, a commonly used preadipocyte cell line, and three-dimension (3D) organoids were obtained^15^. In fact, upon adipogenesis, the organoid size, and lipid and extracellular matrix (ECM) content increased dramatically, and these increases were significantly inhibited in the presence of PGF2α-ags. In our subsequent pilot study, although we also found similar effects of PGF2α-ags toward 3D organoids obtained from human orbital fibroblasts (HOFs), the effect of PGF2α-ags on lipid metabolism during their adipogenesis was much less as compared to the 3D organoid of 3T3-L1 cells^16^. Based upon these findings, we rationally concluded that a 3D tissue culture could replicate DUES pathology reasonably well and therefore should be useful for developing an understanding of the disease etiology of DUES. However, there are still unknown issues that need to be identified for a better understanding of the clinical and pathological aspects of DUES. Among these are 1) the reason why the incidences of DUES are different among PGF2α-ags, 2) which PGF2α-ags affect adipogenesis or other mechanisms, or both, 3) what changes in the physical properties of HOFs are caused by PGF2α-ags.

Therefore, in the current study, to answer above unidentified issues, we further characterized the concentration dependency among PGF2α-ags, the influence of PGF2α-ags toward adipogenesis and gene expression of ECM proteins, as well as their efficacy on the physical properties of the 3D HOFs organoids.

## Methods

This study, which was performed at the Sapporo Medical University Hospital, Japan, was approved by the institutional review board (approved number, 312–3190) and according to the tenets of the Declaration of Helsinki as well as national laws for the protection of personal data. Informed consent was obtained from all participants in this study.

### Two-dimension (2D) cultures of human orbital fibroblasts (HOFs) and induction of their adipogenic differentiation (DIF+)

Initially cultured HOFs preadipocytes prepared from orbital fat explants that were surgically obtained from 2 male and 2 female patients (mean age; 48 years old) with orbital fat herniation^16^. They were further cultured in a 2D growth medium (DMEM supplemented with 10% FBS, 1% L-Glutamine, and 1% Antibiotic–Antimycotic) on 100 mm dishes at 37 °C with 5% CO_2_ in which the medium was changed once every two days until they reached 100% confluence. For the induction of adipogenic differentiation (DIF+) of the 2D cultured HOFs, overconfluent 2D cells were obtained by further culturing for 2 days, and thereafter cultured according to the DIF+ protocol; supplementation of the culture medium with an adipogenesis cocktail (250 nmol/L dexamethasone, 10 nmol/L T3, 10 μg/ml insulin, and 10 mmol/L troglitazone) during Day 1–5, and with the dexamethasone-free adipogenesis cocktail during Day 6–12. Alternatively, as experimental conditions without DIF+ , 2D HOFs cells were assessed as above in the medium used above, supplemented with 0.1% DMSO during the entire 12-day culture period.

### Three-dimension (3D) cultures of HOFs and induction of their adipogenic differentiation in the presence or absence of several PGF2α-ags

For 3D organoid cultures and the induction of DIF+ was performed as described previously^16^. DIF+ and DIF− 3D HOFs organoids were prepared by the same protocols described in the 2D HOFs culture.

For the evaluation of drug efficacy at different concentrations of the PGF2α-ags toward 3D HOFs organoids, latanoprost acid (LAT-A); most popular acid form of prost-type PGF2α-ags and bimatoprost acid (BIM-A); prostamide type PGF2α-ags were selected in addition to PGF2α among the current commercially available PGF2α-ags. At different concentration (0, 1, 100 or 10,000 nmol/L) of each drug was added during Day 1 through 12. These different concentrations used were according to the previous studies^14,16^. These 2D HOFs cells and 3D HOFs organoids were each collected at Day 12 and used for the analytical studies described below.

### Characterization of 3D organoid configuration and measurement of the mean sizes of the 3D HOFs organoids

As described previously, the 3D organoid configuration was observed by phase contrast (PC, Nikon ECLIPSE TS2; Tokyo, Japan)^16^. The mean size of each 3D organoid was defined as the largest cross-sectional area (CSA) of the PC image analyzed using the Image-J software version 1.51n (National Institutes of Health, Bethesda, MD).

### Lipid staining by BODIPY of 2D HOFs cells or 3D HOFs organoids

All procedures were conducted at room temperature. 2D HOFs cells or 3D HOFs organoids obtained at Day 12 as above were washed with phosphate buffered saline (PBS) and then fixed in 4% paraformaldehyde in PBS for 10 min. These organoids were incubated in 0.2% 1:1000 dilutions of BODIPY (488 nm), DAPI and phalloidin (594 nm) in PBS for 1–3 h. Fluorescence intensity of the BODIPY-stained lipid droplets was measured using a Nikon A1 confocal microscope (Tokyo, Japan) and quantified using the Image J software version 2.0.0 (https://imagej.net/Fiji/Downloads, NIH, Bethesda, USA).

### Physical properties of 3D HOFs organoids

To examine the physical properties of the 3D HOFs organoids, the micro-indentation force was measured using a micro-squeezer (MicroSquisher, CellScale, Waterloo, ON, Canada) as described previously^17^. The force required (force/displacement, μN/μm) to compress a single 3D HOFs organoid until a 50% deformity was reached during 20 s was determined.

### Gene expression analysis

Total RNA extraction, reverse transcription and real-time PCR were performed by the methods described previously^16^. After normalization using the housekeeping gene 36B4 (RPLP0), each cDNA quantities are expressed as fold change relative to the control. DNA sequences of primers and TaqMan probes are shown in Supplemental Table 1.

### Immunocytochemistry of 3D HOFs organoids

Immunocytochemistry of the 3D HOFs organoids was conducted by a recently described method^16^ using first antibody; an anti-human COL1 rabbit antibody (1:200 dilutions), second antibody; goat anti-rabbit IgG (1:1000 dilutions, 488 nm), phalloidin (1:1000 dilutions, 594 nm) and DAPI (1:1000 dilutions). Immunofluorescent images (serial-axis 2.2-μm interval images during a z-plane between 35-μm from their surface) were obtained (Nikon A1 confocal microscopy) and their signal intensities were analyzed using Image J (NIS-Elements 4.0 software) as follows: surface area = D × A/(A + π × H^2^), where D (μm) indicates the organoid diameter, A (μm^2^) indicates the area of the sectioned organoid, and H (μm) indicates height (= 35-μm).

### Statistical analyses

All statistical analyses were assessed using GraphPad Prism 7 (GraphPad Software, San Diego, CA). A one-way ANOVA was used, followed by a Tukey's multiple comparison test for evaluation of the difference among matched multiple group comparisons. Data are presented as arithmetic means ± SEM.

## Results

Taketani et al. in a previous study, using a 2D 3T3-L1 culture and FP receptor knockout mice, reported that prost-type PG analogues have the effect of inhibiting adipogenesis through stimulation of the FP receptor^14^. Since orbital fatty tissues grow in a 3D conical space, we developed a 3D drop culture method to establish a more suitable model for replicating DUES etiology^15,16^. Using this method, we found that, in response to either 100 nmol/L BIM-A or 100 nmol/L PGF2α, adipogenesis was suppressed in the 3D 3T3-L1 or HOFs organoids and that ECM expression was altered^15,16^. However, the advantages and disadvantages in our developed 3D DUES models remains to be elucidated. In this study, to further characterize the 3D HOFs DUES model in detail, we investigated some additional clinically and basically informative but unidentified issues; 1) the extent of the difference in adipogenesis between 2 and 3D HOFs, 2) the pharmacokinetics of inducing the 3D organoid downsizing by PGF2α-ags, 3) PGF2α-ags-induced effects toward 3D HOFs preadipocyte organoid (DIF−), 4) the effects of PGF2α-ags on the physical properties of the 3D HOFs organoids, and 5) underlying molecular mechanisms causing PGF2α-ags induced effects.

### Comparison between 2 and 3D cell cultures of HOFs under adipogenic differentiation (DIF+)

**Figure 1:**
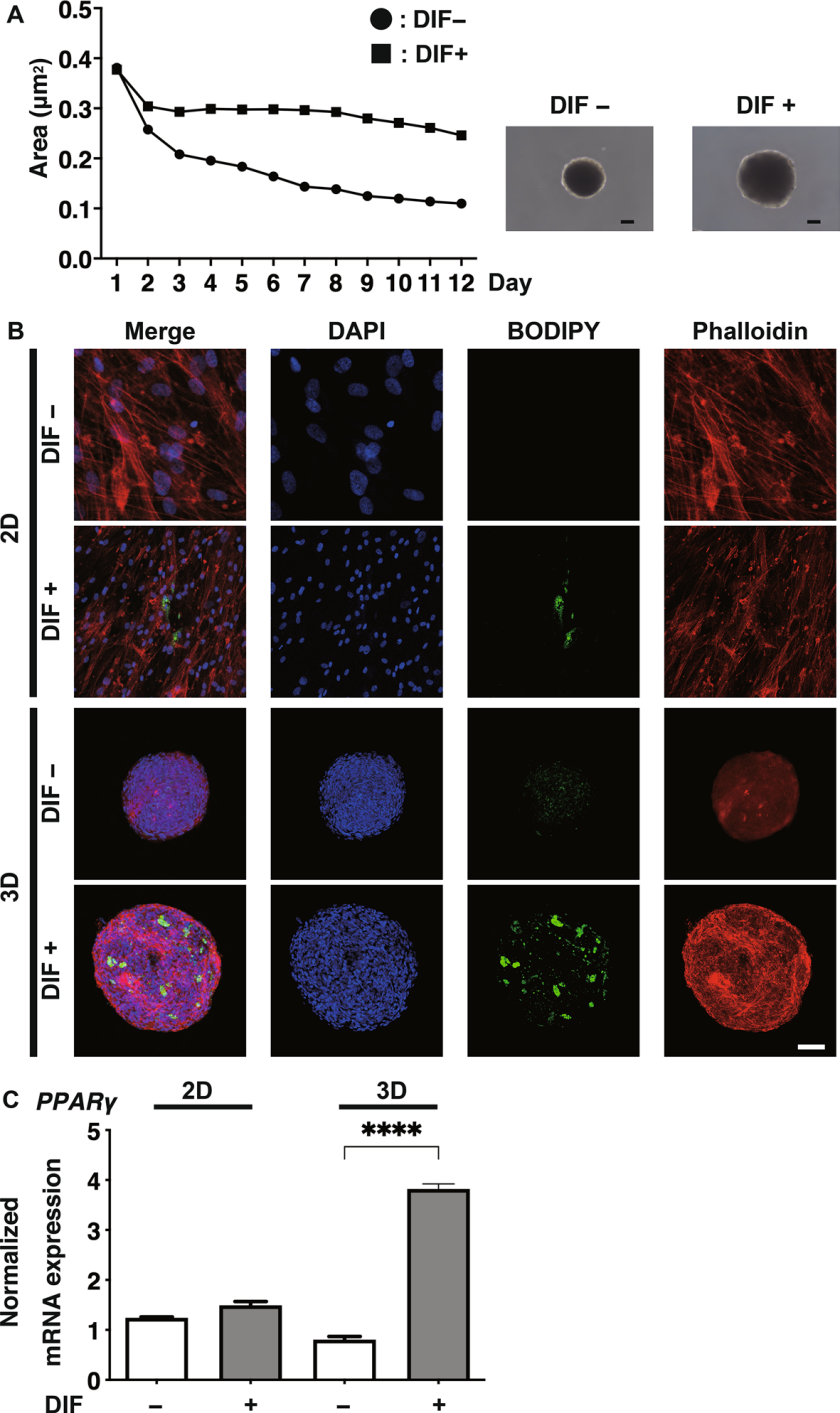
Changes in the sizes of three-dimension (3D) organoids of human orbital fibroblasts (HOFs) during the 3D cell culture, confocal immunostaining images by lipid staining (BODIPY), phalloidin, and DAPI of two-dimension (2D) and 3D cultured HOFs, and mRNA expressions of *peroxisome proliferator-activated receptor γ (PPARγ)* of 2D or 3D cultured HOFs. (Panel **A**) Mean sizes of the 3D organoids of HOFs preadipocytes (DIF−, closed circles) and for adipogenic differentiation (DIF+ , closed squares) were plotted during the 12 days period of the 3D culture, and representative phase contrast images are shown. Scale bar: 100 µm. (Panel **B**): At Day 12, 2D cultured HOFs (DIF− or DIF+) or 3D HOFs organoids (DIF− or DIF+) were immunostained with BODIPY (green), Phalloidin (red) and DAPI (blue). Scale bar: 100 µm. (Panel **C**): mRNA expressions of *PPARγ* of 2D or 3D cultured HOFs (DIF− or DIF+) were plotted. All experiments were performed in duplicate using fresh preparations consisting of 5 organoids each. Data are presented as the arithmetic mean ± the standard error of the mean (SEM). ****P < 0.001 (ANOVA followed by a Tukey’s multiple comparison test).

In order to determine which 2D or 3D HOFs culture is suitable as a DUES model, both cultured HOFs were compared. As shown in Fig. 1 (panel A), the 3D HOFs organoids reproducibly formed uniform round-shaped spheroidal organoids from 20,000 HOFs cells and these organoids gradually grew smaller by Day 12 of their maturation. In addition, the organoids were significantly enlarged upon adipogenic differentiation (DIF+) compared to 3D HOFs preadipocytes (DIF−). Upon DIF+ , positive BODIPY staining in the 2D HOFs was negligible and only a faint staining spot was detected by chance (Fig. 1. panel B). The expression of the *PPARγ* in 2D HOFs cells was not significantly altered (Fig. 1, panel C). While, in contrast, lipid staining with BODIPY (Fig. 1, panel B), and the expression of the *PPARγ* (Fig. 1, panel C) of the 3D HOF organoids were significantly enhanced. These observations indicate that the DIF+ of HOFs was more potently induced in our 3D organoid culture as compared to the conventional 2D culture. We therefore conclude that our 3D HOF organoid culture should be a suitable DUES model and was therefore used in our subsequent investigations as below.

### Effects of different concentrations of PGF2α-ags on the sizes of the 3D HOFs organoids under adipogenic differentiation (DIF+)

**Figure 2:**
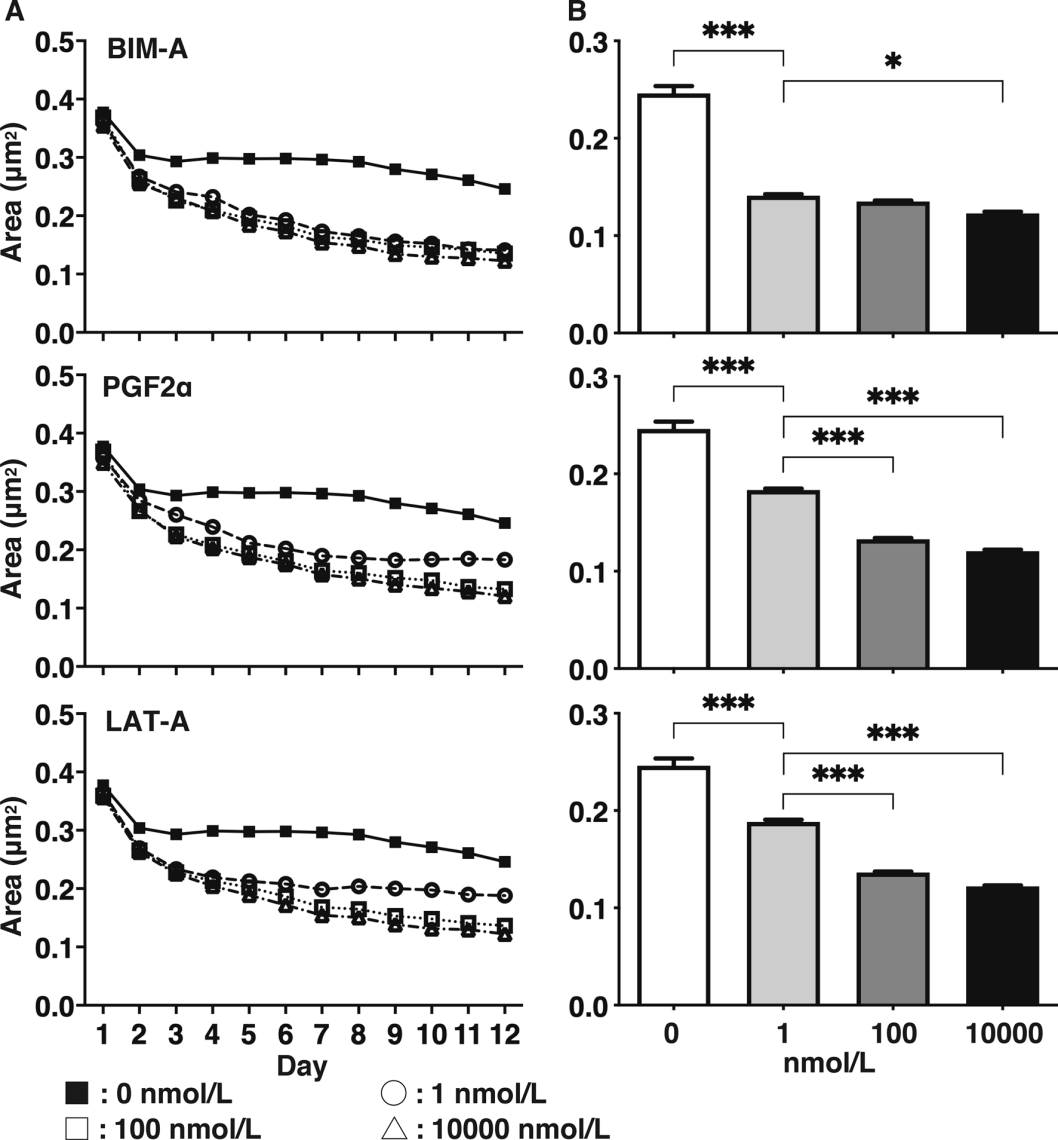
Changes in the sizes of three-dimension (3D) organoids of human orbital fibroblasts (HOFs) during the 3D cell culture in the absence or presence of different concentrations of Prostaglandin F2α analogues (PGF2α-ags). In the absence (closed squares) or presence of 1 (open circles), 100 (open squares) or 10,000 nmol/L (open triangles) of Bimatoprost acid (BIM-A), PGF2α or latanoprost acid (LAT-A), changes in the mean sizes of the HOFs organoids of DIF during the 12-day period of the culture were plotted (panel** A**), and their mean sizes at Day 12 were compared among the different concentrations (panel** B**). All experiments were performed in triplicate using fresh preparations consisting of 16 organoids each. Data are presented as the arithmetic mean ± standard error of the mean (SEM). *P < 0.05, ***P < 0.005 (ANOVA followed by a Tukey’s multiple comparison test).

As shown in Fig. 2, the DIF+ induced enlargement in the HOF organoid sizes was significantly suppressed in the presence of BIM-A, PGF2α or LAT-A and this enlargement was concentration depend (1, 100 or 10,000 nmol/L). Among these PGF2α-ags, BIM-A showed the most potent effects, and the effects of PGF2α and LAT-A were similar. Thus, a subsequent analysis was conducted by using 100 nmol/L BIM-A and 100 nmol/L PGF2α. These differences in drug efficacy among PGF2α-ags were in agreement with clinical observations that BIM most frequently causes DUES, as evidenced by many published reports^9,18^, suggesting that our 3D culture HOFs well replicated the pathogenesis of DUES.

### Effects of PGF2α-ags on the sizes of 3D HOFs organoids preadipocytes (DIF−)

**Figure 3:**
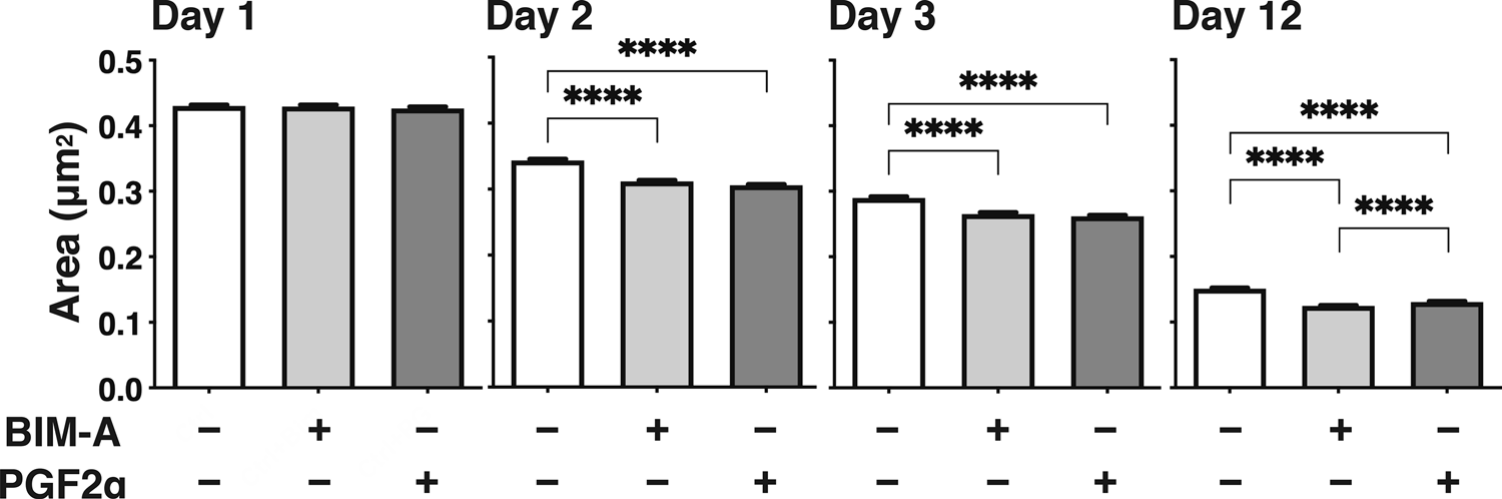
Changes in the sizes of three-dimension (3D) organoids of human orbital fibroblasts (HOFs) preadipocytes (DIF−) during the 3D cell culture in the absence or presence of Prostaglandin F2α analogues (PGF2α-ags). The mean sizes of the organoids of HOFs preadipocytes (DIF−) without or with 100 nmol/L Bimatoprost acid (BIM-A) or 100 nmol/L PGF2α were measured at Day 1, 2, 3 or 12, and compared among the treatment groups. All experiments were performed in triplicate using fresh preparations consisting of 16 organoids each. Data are presented as arithmetic means ± standard error of the mean (SEM). ****P < 0.001 (ANOVA followed by a Tukey’s multiple comparison test).

To elucidate whether these PGF2α-ags-induced effects on the above 3D HOF organoids were dependent on their adipogenesis or not, the effects of PGF2α-ags on the 3D HOF preadipocyte organoids (DIF−) were also examined. As shown in Fig. 3, the downsizing effects toward the DIF− 3D organoids by both 100 nmol/L BIM-A and 100 nmol/L PGF2α were still observed after Day 2 during the 3D culture, although these effects were much less than those with DIF+ , as described in Fig. 2. Taken together, the downsizing effects of the 3D HOFs organoids by PGF2α-ags occurred during the maturation of the 3D HOF preadipocyte organoids themselves and these effects were markedly enhanced upon DIF+ .

### Physical stiffness of the 3D organoid of DIF+ and DIF− HOFs in the absence and presence of PGF2α-ags

**Figure 4:**
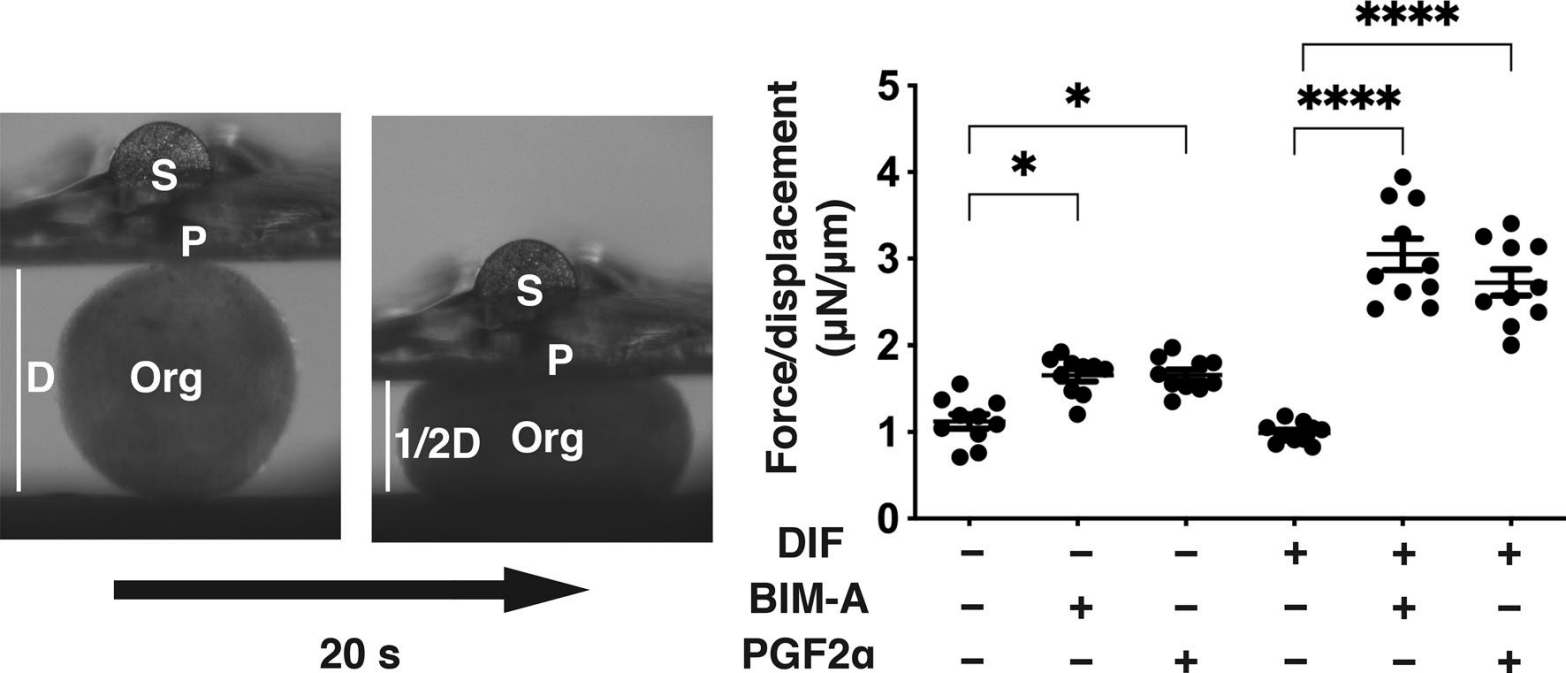
Physical stiffness of three-dimension (3D) human orbital fibroblasts (HOFs) organoids among several experimental conditions. At Day 12, 3D organoids of HOFs preadipocytes (DIF−) and their differentiation (DIF+) in the absence or presence of 100 nmol/L Bimatoprost acid (BIM-A) or 100 nmol/L PGF2α were subjected to the physical property analysis by a micro-squeezer using 10 freshly prepared organoids. A single 3D organoid placed on a 3-mm × 3-mm plate was compressed to 50% deformation during 20 s, those were continuously monitored by a microscopic camera (left panel, S: micro-sensor of the mechanical force (μN), P: compression plate, Org: single 3D organoid,D: the distance across 3D organoid, 20 s: 20 s). Its requiring force was measured, and the force/displacement value (μN/μm) was plotted (right panel). *P < 0.05, ****P < 0.001 (ANOVA followed by a Tukey’s multiple comparison test).

We next examined the physical stiffness of the 3D HOFs organoids by means of a micro-squeezer analysis^19,20^ under several conditions, as described above. Significantly higher mechanical indentation forces were required for the organoids in the presence of PGF2α-ags, and these PGF2α-ags-induced effects were further enhanced upon DIF+ (Fig. 4).

### Comparison between 3D organoid sizes of DIF+ and DIF− HOFs, and the mRNA expression of adipogenesis-related genes, ECMs and TIMPs

**Figure 5:**
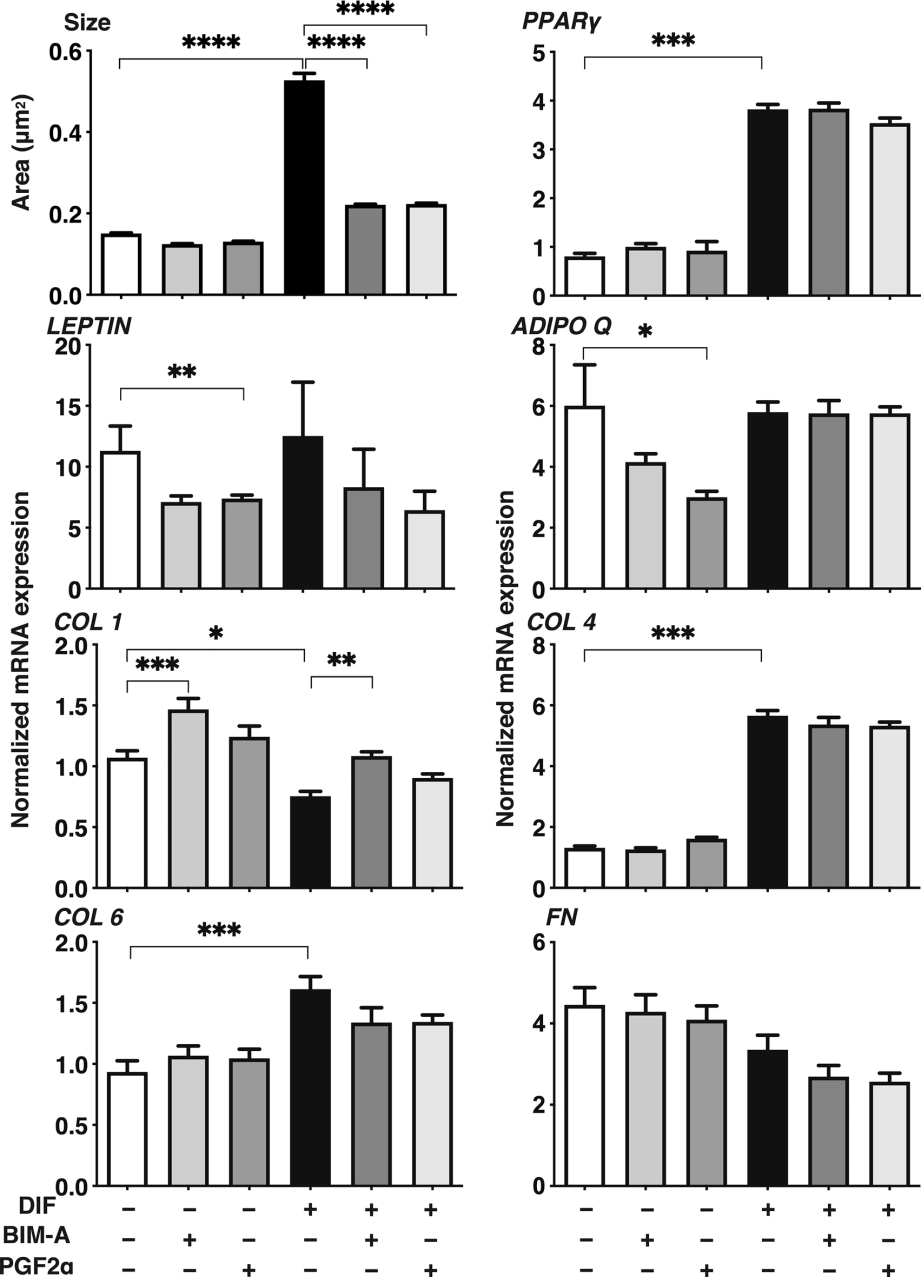
Comparison between the mean sizes of three-dimension (3D) human orbital fibroblasts (HOFs) organoids and their gene expressions of adipogenesis related genes and extracellular matrix (ECM) among several experimental conditions. At Day 12, 3D organoids of HOFs preadipocytes (DIF−) and their differentiation (DIF+) in the absence or presence of 100 nmol/L Bimatoprost acid (BIM-A) or 100 nmol/L PGF2α were subjected to qPCR analysis to estimate the mRNA expression of adipogenesis related genes (*PPARγ*: peroxisome proliferator-activated receptor γ, *LEPTIN*, *ADIPO Q*) and ECM (*COL1*: collagen1, *COL4*: collagen4, *COL6*: collagen6, *FN*: fibronectin). The values were compared with the mean sizes of the 3D HOF organoids among several treatment groups as shown in Figs. 1, 2, 3. All experiments were performed in duplicate using fresh preparations consisting of 4 organoids each. Data are presented as the arithmetic mean ± standard error of the mean (SEM). *P < 0.05, **P < 0.01, ***P < 0.005, ****P < 0.001 (ANOVA followed by a Tukey’s multiple comparison test).

**Figure 6:**
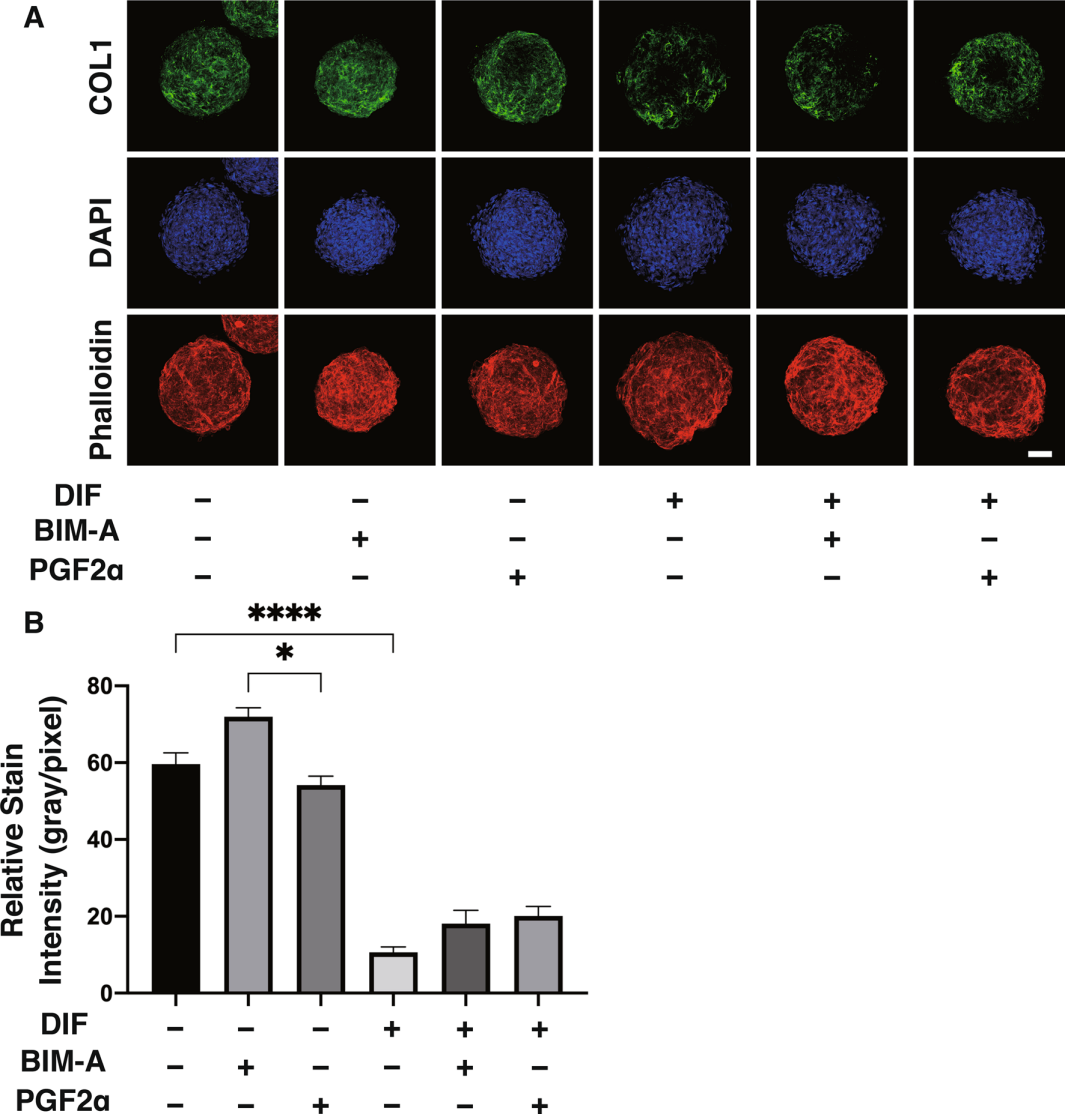
Representative confocal immunofluorecein images of the expression of collagen1 (COL1) in three-dimension (3D) human orbital fibroblasts (HOFs) organoids. At Day 12, 3D culture organoids of HOF preadipocytes (DIF−) and their differentiation (DIF+) in the absence or presence of 100 nmol/L Bimatoprost acid (BIM-A) or 100 nmol/L PGF2α were immunestained with specific antibodies of COL1 (collagen1, green), DAPI (blue) and phalloidin (red). Scale bar: 100 µm. Representative confocal images were shown in (panel **A**), and their staining intensities toward COL1 were plotted in (panel **B**). All experiments were performed in duplicate using fresh preparations consisting of 5 organoids each (total 10 organoids). Data are presented as the arithmetic mean ± standard error of the mean (SEM). *P < 0.05, ****P < 0.001 (ANOVA followed by a Tukey’s multiple comparison test).

**Figure 7:**
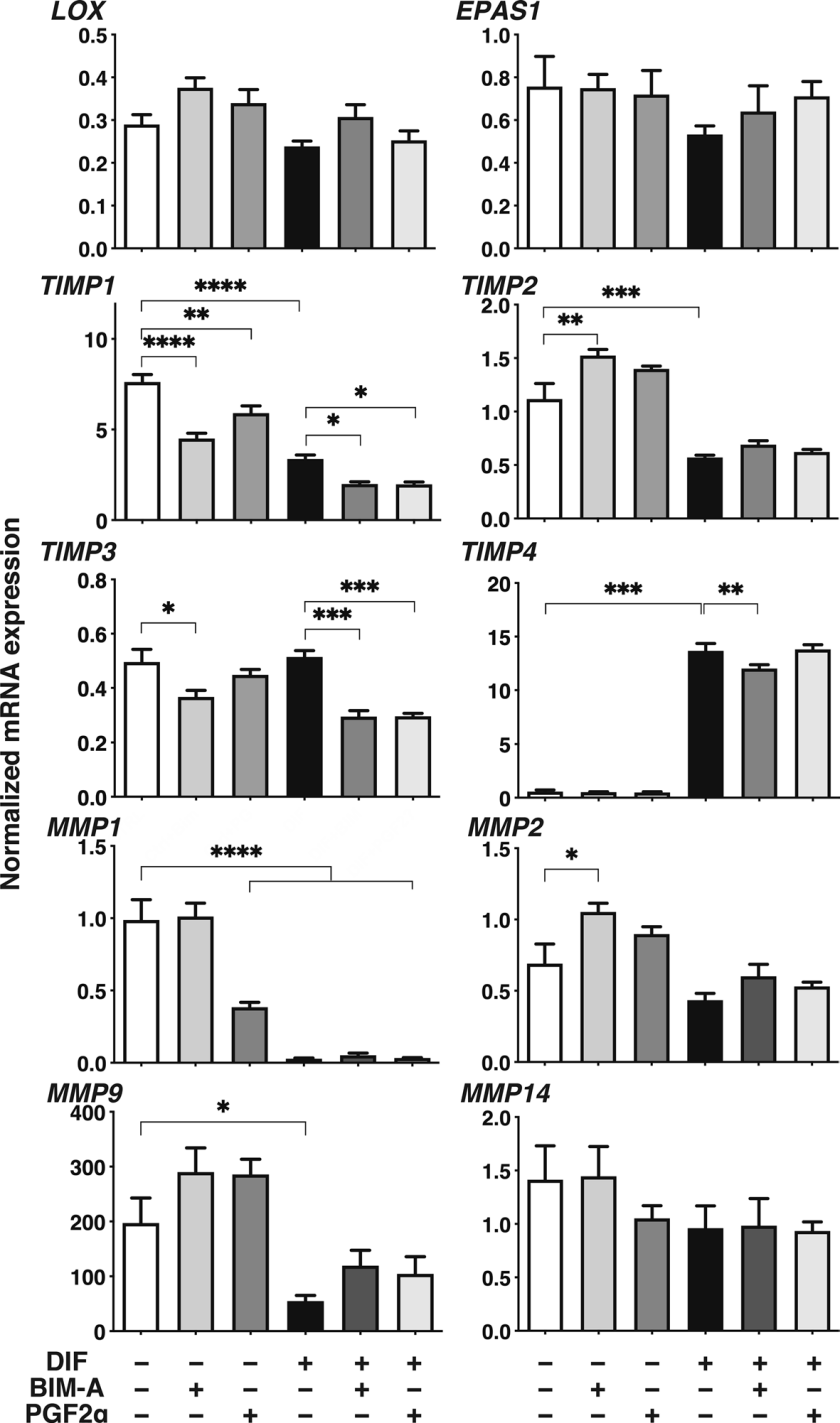
mRNA expression of ECM regulatory genes, tissue inhibitors of metalloproteinases (TIMPs), and matrix metalloproteinases (MMPs) among several experimental conditions. At Day 12, 3D organoids of 3D HOFs preadipocytes as the control (DIF−) and their differentiation (DIF+) in the absence or presence of 100 nmol/L Bimatoprost acid (BIM-A) or 100 nmol/L PGF2α were subjected to qPCR analysis to estimate the mRNA expression of ECM regulatory genes including *lysyl oxidase* (*LOX)* and *endothelial Per-Arnt-Sim domain-containing protein 1* (*EPAS1)*, tissue inhibitors of metalloproteinases 1–4 *(TIMP1-4),* and matrix metalloproteinases (*MMP1, 2, 9* and *14*). All experiments were performed in duplicate using fresh preparations consisting of 4 organoids each. Data are presented as the arithmetic mean ± standard error of the mean (SEM). *P < 0.05, **P < 0.01, ***P < 0.005, ****P < 0.001 (ANOVA followed by a Tukey’s multiple comparison test).

To elucidate the underlying molecular mechanisms responsible for these effects by PGF2α-ags as above, under a variety of conditions of DIF− or DIF+ and in the absence or presence of PGF2α-ags, changes in the sizes of 3D HOFs organoids were compared to the mRNA expression of several factors associated with adipogenesis related genes (*PPARγ*, *LEPTIN* and *ADIPO Q*), and ECM (*COL1, COL4, COL6* and *FN*) (Fig. 5). Among the factors tested, a significant reverse correlation between 3D HOF organoid sizes and mRNA expression of *COL1* was observed. This reverse correlation was also observed by immunostaining for COL1 (Fig. 6). To study this issue further, since the related factors that influence the metabolism of ECMs including COL1, the mRNA expression of ECM regulatory genes (*LOX* and *EPAS1*), tissue inhibitors of metalloproteinase 1–4 (*TIMP 1–4*), and matrix metalloproteinase (*MMP*) 1, 2 9 and 14 were examined (Fig. 7). The above genes were selected among several ECM regulatory genes that were pivotally related to the fibrosis of human adipocytes^21^ or Graves’ orbitopathy (GO) related HOFs^19^. Interestingly, among these, the fluctuations in the expression of the mRNA of *TIMP2,* and *MMPs 2 and 9* under several conditions shown in Fig. 7 also similarly corresponded with the values for *COL1*, as shown in Fig. 5. In addition, the profiles for LOX were also similar to that for *COL1*, although then were not statistically significant because of the different experimental conditions that were used. Furthermore, the expression of *TIMPs 1, 2* and *4*, and *MMPs 1* and *9* were modulated upon DIF+ , and those for T*IMPs 1* and *3*, and *MMP 2* may also be affected by PGF2α-ags. Taken together, as possible underlying mechanisms of DIF+ and PGF2α-ags induced effects toward the sizes of the 3D HOF organoids, it therefore appears that *COL1, TIMP2* and *MMPs 2* and *9* may be related.

## Discussion

Different incidents of DUES have been reported among the PGF2α-ags that are clinically used as anti-glaucoma medications; 60.0% in patients using BIM, 50.0% in those using travoprost, 24.0% in those using LAT, and 18.0% in those using tafluprost^22^. Miki et al. reported that DUES was observed before trabeculectomy procedures in 18 of 74 eyes (24.3%) in patients that had been treated with BIM (9 eyes; 50.0%), LAT (3 eyes; 16.7%), tafluprost (1 eye; 5.5%) and travoprost (5 eyes; 27.8%)^23^. Furthermore, they also reported that no significant recurrent IOP elevation at 24 months post-trabeculectomy was found (P < 0.001) in patients using BIM (31.3%) than LAT (83.2%), tafluprost (45.5%), or travoprost (65.6%), as well as being significantly (P < 0.0001) lower in patients with DUES (34.7%) compared to those without DUES (74.3%). Based upon these observations, the authors suggested that BIM may result in an unfavorable post-trabeculectomy outcome in addition to inducing a high incidence of DUES. In the current study of the concentration dependency of PGF2α-ags using 3D HOF organoids, we also demonstrated that BIM-A has the most potent efficacy among the PGF2α-ags for the suppression of DIF+ induced enlargement in the 3D HOF organoids.

Adipocytes are involved in a variety of physiological functions related to fat storage and the mobilization of free fatty acids in response to a variety of nutritional and hormonal conditions^24^. During their DIF+ , preadipocytes grow into the mature form of adipocytes and this process is minutely regulated by several adipogenesis-related genes such as *PPARγ*^25,26^*.* In terms of the effects of PGF2α-ags toward DIF+ , it was reported that PGF2α-ags, including BIM, travoprost, LAT, and tafluprost suppressed preadipocyte growth^14,27^. However, in contrast, the effects of PGF2α-ags toward preadipocytes (DIF−) have not yet been elucidated so far. In the current study, PGF2α-ags were also observed to have a significant effect on 3D HOFs DIF− organoids. That is, PGF2α-ags results in downsized and harder 3D HOF DIF− organoids, and the up-regulation of the mRNA expression of the *COL1, TIMP2*, and *MMPs 2* and *9* genes was induced, although these effects were quite similar but less than the corresponding values found for 3D HOFs DIF+ organoids. Alternatively, it was revealed that functionally active FP receptors are located on human trabecular meshwork (TM) cells^28^, and, as a result, PGF2α-ags could affect TM outflow of the aqueous humor^29,30^. Taken together with the effects of PGF2α-ags on trabeculectomy outcomes, as alluded to above, PGF2α-ags may affect mechanisms other than DIF+ , such as ECM metabolism, even in adipocyte tissues.

Compared to conventional 2D cell cultures, the 3D organoid culture method is more representative for studies of the structure of tissues in a closed biological environment including the network of ECM proteins^31^. Thus, the 3D organoid culture system has attracted substantial interest in terms of serving as ex vivo disease models for developing an understanding of their disease etiology as well as the design of therapeutic strategies^32^. In the present study, we report that the efficacy of DIF+ toward HOFs was much higher in 3D organoid cultures than in conventional 2D cell cultures. In terms of this difference between 2 and 3D cell cultures, we speculate that DIF+ may prefer a 3D environment. In fact, Miyamoto et al. also reported a much higher efficacy of DIF+ of human adipose-derived stem cells in 3D cell cultures than in 2D cell cultures^33^. In addition, our group, in a study using 3D organoid cultures, also recently found that the Endothelial Per-Arnt-Sim domain protein 1 (EPAS1) encoding hypoxia-inducible factor-2A (HIF2A) is pivotally involved in mediating LOX-dependent ECM accumulation, resulting in a significant enhancement in the levels of orbital fat in patients with GO. However, in contrast, these EPAS1 effects were not observed in conventional 2D cell cultures^19^.

It is well known that ECM provides not only structural support for organs, but also functions to modify and regulate cell–cell signaling as well as various other cellular functions^34^. Among the ECM molecules, COLs, which are especially fibrillar collagen molecules, are located essentially apart from cells, not only at the cell-ECM interface, and among the 28 distinct collagen types, fibrillar type I collagen is the most abundant type^35^. In the present study, the physical stiffness of the 3D HOFs organoids were significantly increased in parallel with the PGF2α-ags-induced up-regulation of COL1. Similarly, the COL1 induced enhancement of fibrosis was reported in a murine bleomycin-induced pulmonary fibrosis model induced by an FP agonist^36–38^. LOX and four LOX-like proteins are members of a family of copper-containing amine oxidases, and play a role in the oxidation of lysine and hydroxylysine residues of ECMs, such as COLs and elastin, as well as other soluble substrates^39,40^. In addition to LOX, EPAS1 is also involved in the regulation of ECM remodeling in orbital adipose tissue fibrosis in GO^19^. In fact, EPAS1 was identified as a pivotal regulator of the LOX-induced remodeling of the ECM and tissue stiffness^41^. However, in the present study, the mRNA expression of EPAS1 was not significantly altered upon adipogenesis and/or in cases of the administration of PGF2α-ags. Since our HOFs were obtained from non-inflammatory orbital fat tissues, it is possible that they are different from those from patients with GO. Alternatively, MMP proteins are known to be major proteases that are involved in ECM degradation^42^, and their proteolytic properties are regulated during the activation of the inactive proenzymes (pro-MMP) by tissue inhibitors of metalloproteinases (TIMPs)^43^. In the present study, DIF+ and/or PGF2α-ags were round to induce alterations in the sizes of the 3D HOFs organoids and these alterations appear to be correlated inversely with the the mRNA expression of *TIMP2*, and *MMPs 2* and *9*, in addition to *COL1*. Furthermore, the mRNA expression of *TIMPs 1, 2* and *4*, and *MMPs 1* and *9*, and that for T*IMPs 1* and *3*, and *MMP 2* were also altered by DIF+ and PGF2α-ags, respectively, suggesting that other currently unknown mechanisms may well be associated with this process. Thus, to elucidate the molecular mechanisms responsible for causing these TIMPs and MMPs upon DIF+ as well as PGF2α-ags, additional investigations using specific inhibitors and siRNA toward possible upstream signaling molecules will be our next focus.

## Supplementary Information

Supplementary Information
