# Supplementary material for: Prostaglandin F2α agonists induced enhancement in collagen1 expression is involved in the pathogenesis of the deepening of upper eyelid sulcus
Source: Sci Rep. 2021 Apr 26;11:9002. doi: 10.1038/s41598-021-88562-4 (PMC8076191; doi:10.1038/s41598-021-88562-4)
Supplement: Supplementary file 1 — Supplementary Information [file 41598_2021_88562_MOESM1_ESM.pdf]

Prostaglandin F2α agonists induced enhancement in collagen1 expression is involved in the pathogenesis of the deepening of upper eyelid sulcus

Key words: deepening of the upper eyelid sulcus (DUES), prostaglandin (PG) analogues, 3-dimension (3D) tissue culture

Kaku Itoh, Yosuke Ida, Hiroshi Ohguro, Fumihito Hikage.

Departments of Ophthalmology, Sapporo Medical University School of Medicine

All correspondence should be addressed to Fumihito Hikage

Tel# 81-11-611-2111, Fax# 81-11-613-6575, e-mail: fuhika@gmail.com

Both authors (K. I and Y. I.) contributed equally to this study.

		Sequence	Exon Location	RefSeqNumber
human RPLP0	Probe	5’-/56-FAM/CCCTGTCTT/ZEN/CCCTGGGCATCAC/3IABkFQ/-3’	2-3	NM_001002
	Forward	5’-TCGTCTTTAAACCCTGCGTG-3’		
	Reverse	5’-TGTCTGCTCCCACAATGAAAC-3’		
human PPARγ	Probe	5’-/56-FAM/CTCATAATG/ZEN/CCATCAGGTTTGGGCG/3IABkFQ/-3’	7-8	NM_138712
	Forward	5’-GTTTCAGAAATGCCTTGCAGT-3’		
	Reverse	5’-GGATTCAGCTGGTCGATATCAC-3’		
human FABP4	Probe	5’-/56-FAM/CAGGAAAGT/ZEN/GGCATGGC/3IABkFQ/-3’	1-2	NM_001442
	Forward	5’-ACTTGTCTCCAGTGAAAACTTTG-3’		
	Reverse	5’-ATCACATCCCCATTCACACT-3’		
human ADIPOQ	Forward	5’-AGCATCCTGAGCCCTGAT-3’	1-3	NM_00177800
	Reverse	5’-CCTCACTTCCATTCTGACTGC-3’		
human LEPTIN	Forward	5’-AGAGTGGCTTAGAGGAGTCAG-3’	3-3	NM_000230
	Reverse	5’-TGGCTTCCAGGTATCTCCA-3’		
human COL1A1	Probe	5’-/56-FAM/TCGAGGGCC/ZEN/AAGACGAAGACATC/3IABkFQ/-3’	1-2	NM_000088
	Forward	5’-GACATGTTCAGCTTTGTGGAC-3’		
	Reverse	5’-TTCTGTACGCAGGTGATTGG-3’		
human COL4A1	Probe	5’-/56-FAM/TCATACAGA/ZEN/CTTGGCAGCGGCT/3IABkFQ/-3’	51-52	NM_001845
	Forward	5’-AGAGAGGAGCGAGATGTTCA-3’		
	Reverse	5’-TGAGTCAGGCTTCATTATGTTCT-3’		
human COL6A1	Forward	5’-CCTCGTGGACAAAGTCAAGT-3’	2-3	NM_001848
	Reverse	5’-GTGAGGCCTTGGATGATCTC-3’		
human FN1	Forward	5’-CGTCCTAAAGACTCCATGATCTG-3’	3-4	NM_212482
	Reverse	5’-ACCAATCTTGTAGGACTGACC-3’		
human TIMP1	Probe	5’-/56-FAM/TCAACCAGA/ZEN/CCACCTTATACCAGCG/3IABkFQ/-3’	2-4	NM_003254
	Forward	5’-CCTTCTGCAATTCCGACCT-3’		
	Reverse	5’-GCTTGGAACCCTTTATACATCTTG-3’		
human TIMP2	Probe	5’-/56-FAM/TCTCATTGC/ZEN/AGGAAAGGCCGAGG/3IABkFQ/-3’	3-4	NM_003255
	Forward	5’-GACGTTGGAGGAAAGAAGGA-3’		
	Reverse	5’-TGTGGTTCAGGCTCTTCTTC-3’		
human TIMP3	Probe	5’-/56-FAM/CCTCCTTTA/ZEN/CCAGCTTCTTCCCCAC/3IABkFQ/-3’	1-3	NM_000362
	Forward	5’-CCTTCTGCAACTCCGACATC-3’		
	Reverse	5’-CGGTACATCTTCATCTGCTTGA-3’		
human TIMP4	Probe	5’-/56-FAM/ACTGAGGAC/ZEN/CTGACCAGTCAAGAGA/3IABkFQ/-3’	3-4	NM_003256
	Forward	5’-GGTTTGAGAAAGTCAAGGATGTTC-3’		
	Reverse	5’-GTTGCACAGATGGATGAAGAC-3’		
human LOX	Forward	5’-ACATTCGCTACACAGGACATC-3’	6-7	NM_002317
	Reverse	5’-TTCCCACTTCAGAACACCAG-3’		
human EPAS1	Forward	5’-AGCCTATGAATTCTACCATGCG-3’	7-8	NM_001430
	Reverse	5’-CTTTGCGAGCATCCGGTA-3’		

Supplemental Table 1. DNA sequences of primers and TaqMan probes are shown.
